# Maize growth promotion by inoculation with *Azospirillum brasilense* and metabolites of *Rhizobium tropici* enriched on lipo-chitooligosaccharides (LCOs)

**DOI:** 10.1186/s13568-015-0154-z

**Published:** 2015-11-14

**Authors:** Bettina Berquó Marks, Manuel Megías, Francisco Javier Ollero, Marco Antonio Nogueira, Ricardo Silva Araujo, Mariangela Hungria

**Affiliations:** Embrapa Soja, C.P. 231, Londrina, Paraná 86001-970 Brazil; Depto de Microbiología, Facultad de Biología, Universidad de Sevilla, Avda. Reina Mercedes 6, Apdo Postal 41012 Seville, Spain; Total Biotecnologia Indústria e Comércio S/A, Rua Emílio Romani 1190, CIC, Curitiba, Paraná 81460-020 Brazil

**Keywords:** Nod factor, *Zea mays*, Inoculant, PGPB

## Abstract

There is an increasing interest in the development and use of inoculants carrying plant growth-promoting bacteria (PGPB) in crops of agronomic interest. The great majority of the inoculants commercialized worldwide contain rhizobia for legume crops, but the use of PGPB as *Azospirillum* spp. for non-legume is expanding, as well as of inoculants combining microorganisms and microbial metabolites. In this study we evaluated the effects of inoculants containing *Azospirillum brasilense* with or without metabolites of *Rhizobium tropici* strain CIAT 899 highly enriched in lipo-chitooligosaccharides (LCOs) in six field experiments performed for three summer crop seasons in Brazil with maize (*Zea mays* L.). Inoculants and metabolites were applied either at sowing by seed inoculation, or by leaf spray at the V3 stage of plant growth. Improvement in shoot dry weight (SDW) and total N accumulated in shoots (TNS) by single, but especially by dual inoculation was observed in some of the experiments. Statistically significant increases in grain yield in relation to the non-inoculated control were observed in five out of six experiments when maize was inoculated with *Azospirillum* supplied with enriched metabolites of *R. tropici* applied by seed or leaf spray inoculation. The results give strength to the development of a new generation of inoculants carrying microorganisms and microbial molecules.

## Introduction

Inoculation of crops of agronomic interest with plant growth-promoting bacteria (PGPB)—especially those belonging to the group of rhizobia associated with legumes—represents a biotechnological practice consolidated worldwide (Bashan et al. 2014). In Brazil, for example, about 25 million doses of inoculants are commercialized every year, 95 % of which for the soybean (*Glycine max* [L.] Merr.) crop (Hungria and Mendes 2015).

The symbiotic interaction between rhizobia and the host legumes to establish the biological nitrogen fixation process involves an intense exchange of signals between the partners. The dialogue starts with the exudation of molecules by the plant—especially flavonoids—which act as signals to the rhizobia (Hungria et al. 1992; Hungria and Phillips 1993; de Rijke et al. 2006). The interaction occurs by means of a key protein in rhizobia—NodD—launching the expression of all other bacterial nodulation genes; in the following step, rhizobia reply with the synthesis and secretion of Nod Factors (Phillips 2000). Nod Factors are lipo-chitooligosaccharides (LCOs), which may comprise up to 60 different structural arrangements (D’Haeze and Holsters 2002), depending on the bacterial species and the environmental conditions (Folch-Mallol et al. 1996; Debellé et al. 2003; Estévez et al. 2009; del Cerro et al. 2015a, b). LCOs act directly in root colonization and cortex cell division (Spaink et al. 1998; Dardanelli et al. 2008).

Intriguingly, it has been reported that LCOs can also promote growth of non-leguminous plants, one possible explanation being because they mimic the effects of plant hormones such as cytokinins and auxins (Rélic et al. 1993), resulting in increased seed germination and resistance to pathogens (Miransari and Smith 2009). With the current knowledge about the effects of LCOs, a new generation of inoculants based on, or enriched with LCOs has proven to be very effective with legumes, and is now advancing to the use with non-legumes (Marks et al. 2013; Smith et al. 2015).

The technology of inoculation of non-legumes with non-symbiotic PGPB—whose main representative is *Azospirillum* spp.—is also being increasingly adopted in several countries, especially for crops such as maize (*Zea mays* L.) and wheat (*Triticum aestivum* L.) (Díaz-Zorita and Fernandez-Canigia 2009; Hartmann and Bashan 2009; Smith et al. 2015). In Brazil, inoculants containing *Azospirillum brasilense* strains Ab-V5 and Ab-V6 were exponentially employed by farmers in the past 5 years for maize and wheat (Hungria et al. 2010; Hungria 2011). More recently, co-inoculation of soybean with rhizobia and azospirilla has also been adopted as an agronomic practice by several farmers, with reported positive effects on nodulation precocity (Chibeba et al. 2015) and increases in grain yield (Hungria et al. 2013, 2015b); positive effects on common bean (*Phaseolus vulgaris* L.) yield have also been reported (Hungria et al. 2013). Among the benefits of inoculation with non-symbiotic PGPB, the contributions of biological nitrogen fixation (Ashraf et al. 2011), production of phytohormones (Strzelczyk et al. 1994), phosphate solubilization (Rodriguez et al. 2004) and control of plant pathogens (Araujo et al. 2005; Wang et al. 2009) are commonly cited.

Studies of the application of LCOs associated with *Azospirillum* to cereals crops are still incipient. Our research group has previously reported that the addition of concentrated metabolites (CM) from two strains of rhizobia containing LCOs resulted in significant increases in maize grain yield (Marks et al. 2013). *Rhizobium tropici* is a very interesting species that synthesizes a wide variety of LCOs, even in the absence of plant inducers (Estévez et al. 2009; del Cerro et al. 2015a, b), making it an interesting bacterium for metabolite production. In this study, metabolites of *R. tropici* strain CIAT 899 highly enriched in LCOs were obtained and applied along with *A. brasilense* in six field experiments performed in 3 years, aiming at getting a better understanding of the combined effects *Azospirillum* and rhizobial molecules on the growth and yield of cereals.

## Materials and methods

### Inoculant and lipo-chitooligosaccharides (LCOs) preparation

Liquid inoculants were prepared with *A. brasilense* strains CNPSo 2083 (=Ab-V5) and CNPSo 2084 (=Ab-V6). Strains are deposited in the Diazotrophic and Plant Growth Promoting Bacteria Culture Collection of Embrapa Soja (WFCC Collection #1213, WDCM Collection #1054). These two elite strains were identified in a previous selection program for the maize and wheat crops (Hungria et al. 2010; Hungria 2011) and are used in commercial inoculants in Brazil. Inoculant concentration was determined by spread-plating on NFb (Hungria and Araujo 1994; Döbereiner et al. 1995) and RC (Cassán et al. 2010) solid media and adjusted to the concentration of 2 × 10^8^ CFU (colony forming units) mL^−1^ in all three crop seasons.

Metabolites of *R. tropici* strain CIAT 899 enriched in LCOs were produced with a combination of procedures. Bacterium growth and extraction of the supernatant *n*-butanol were performed as described by Sanjuan et al. (1992). Purification was accomplished by solid-phase chromatography, with SPE C18 Resprep, Teknokroma column, concentration and lyophilization were performed as described by Soria-Díaz et al. (2003) and Guasch-Vidal (2011). Prior to sowing, lyophilized metabolites of *R. tropici* CIAT 899 were re-suspended in a mixture of acetonitrile and water (20 %). The concentration was adjusted to 0.1 mL L^−1^, corresponding to approximately 10^−9^ M. The metabolites were added to the inoculant containing *Azospirillum* at the time of inoculation, either when applied to the seeds, or by spraying.

### Field experiments

#### Sites description

Six field experiments were conducted over a 3-year period, always in the summer crop season. Two experiments were conducted in 2012/2013, in Ponta Grossa, State of Paraná (southern region) and Cachoeira Dourada, State of Goiás (central-western region), two others in 2013/2014, in Rio Verde, State of Goiás (central-western region), and Maracaí, State of São Paulo (southeastern region), and two others in the crop season of 2014/2015, in Londrina, State of Paraná (southern region) and Ponta Grossa.

Ponta Grossa (25°13′S, 50°1′W) is at 880 m of altitude and has a Köppen-Geiger climate type *Cfb* (temperate with mild summer). Cachoeira Dourada (18°29′S; 49°28′W) is at 450 m of altitude and has a climate type *Aw* (tropical with dry season in the winter). Rio Verde (17°47′S; 50°54′W) is at 730 m altitude and has a climate type *Aw*, Maracaí (22°36′S; 50°40′O) is at 475 m and has a climate type *Cfa* (tropical humid with warm summer) and Londrina (23°11′S, 51°11′W) is at 620 m altitude and has a climate type *Cfa*. The trials were performed on soils classified as Latossolo Vermelho Distrófico (Brazilian classification) (Typic Hapludox, USA Soil Taxonomy) (Ponta Grossa, Cachoeira Dourada, Rio Verde, Maracaí), and Latossolo Vermelho Eutroférrico (Brazilian classification) (Rhodic Eutrudox, USA Soil Taxonomy) in Londrina.

At each site, 2 months before the experiments were established twenty soil samples (0–20 cm depth) were taken to evaluate chemical properties, granulometry and biological properties. For chemical analyses, the samples were previously dried (60 °C for 48 h), sieved (2 mm), and analyzed as described before; soil granulometry was also analyzed as described before (Hungria et al. 2010; Hungria et al. 2015a). Population of free-living diazotrophic bacteria was estimated by the NMP method with dilutions in NFb semi-solid medium (Hungria and Araujo 1994; Döbereiner et al. 1995). Soil properties are shown in Table 1.

**Table 1:** Soil chemical properties, granulometry and population of free-living diazotrophic bacteria at the 0–20 cm layer of the field sites before sowing

Site	pH (CaCl_2_)	Al (cmol_c_ dm^−3^)	H + Al (cmol_c_ dm^−3^)	K (cmol_c_ dm^−3^)	Ca (cmol_c_ dm^−3^)	Mg (cmol_c_ dm^−3^)	P (g dm^−3^)	C (g dm^−3^)	SB (cmol_c_ dm^−3^)	BS	Granulometry (%) MPN^a^			
										%	Clay	Silt	Sand	NMP soil g^−1^
2012/2013														
Ponta Grossa	4.60	0.26	3.07	0.37	3.55	1.73	1.71	18.55	5.65	64.79	58.4	15.7	25.9	1.5 × 10^4^
Cachoeira Dourada	5.40	0.00	7.89	0.15	2.02	1.30	0.80	30.50	3.47	30.54	57.8	18.2	24.0	4.5 × 10^6^
2013/2014														
Rio Verde	5.02	0.10	3.03	0.17	3.46	0.94	2.45	29.43	4.57	60.13	36.3	9.6	54.1	2.5 × 10^6^
Maracaí	5.42	0.00	1.12	0.05	1.20	0.34	6.57	4.88	1.59	58.67	8.8	0.8	90.4	1.5 × 10^6^
2014/2015														
Londrina	5.20	0.00	3.67	0.50	3.91	1.75	11.10	11.10	6.16	62.66	71.0	8.2	20.8	9.0 × 10^6^
Ponta Grossa	4.90	0.07	4.10	0.44	2.95	1.06	25.70	18.00	4.45	52.05	23.8	3.0	73.2	4.5 × 10^5^

*SB* sum of bases, *BS* bases saturation = [(K + Ca + Mg)/T_cec_] × 100, where T_cec_ = K + Ca + Mg + total acidity at pH 7.0 (H + Al)

^a^Estimated by the most probable number method by dilutions and counts in semi-solid NFb medium

About 50 days before starting the experiment, lime was applied to alleviate acidity when necessary, based on soil pH values. The amount of lime applied was estimated for a base saturation of 50 %, to increase the pH to 5.5 or higher.

### Treatments, experimental design and field management

The maize hybrids used in the experiments were DOW 2B 707 HX (Dow AgroSciences) in 2012/2013, DKB-350-PRÓ (Dekalb) in 2013/2014, and DKB-350-PRÓ2 (Dekalb) in 2014/2015. Seeds were not surface disinfected.

Two methods for the inoculation with *A. brasilense* strains CNPSo 2083 and CNPSo 2084 were tested. The first method consisted of seed inoculation at sowing and the second of leaf spray with the same inoculant at the V3 stage (third leave developed).

The experiments consisted of five treatments: (1) non-inoculated control (NI); (2) seed inoculation (SI) with *Azospirillum* at sowing; (3) SI + metabolites enriched with LCOs applied at sowing; (4) leaf spray inoculation (LSI) with *Azospirillum* strains at V3 stage; (5) LSI + enriched metabolites at the V3 stage.

Seed inoculation was performed at a rate of 100 mL 20 kg^−1^ of seeds (2 × 10^8^ CFU mL^−1^) while the spray inoculation was applied at a rate of 200 mL ha^−1^ (2 × 10^8^ CFU mL^−1^) diluted in 100 L of water; 20 kg of seeds give rise to a population of about 60,000 plants ha^−1^. Enriched metabolites were prepared at a concentration of 0.1 mL L^−1^ and mixed with the inoculant before application at a rate of 50 mL 20 kg^−1^ of seeds when applied to the seeds and of 100 mL ha^−1^ when sprayed.

As mentioned above, the main objective of our study was to verify the plant growth-promoting activity of the enriched bacterial metabolites. Therefore, all treatments received the same fertilization, consisting of 300 kg ha^−1^ of a formulation of 08-20-20 (corresponding to 24 kg of N, 60 kg of P and 60 kg of K ha^−1^) at sowing and a topdressing fertilization at the V4 stage (four fully developed leaves), representing 75 % of the usual dose of N-fertilizer recommended for the crop in Brazil, corresponding to 90 kg N ha^−1^ of urea (67.5 kg N ha^−1^).

Plots measured 4.5 m (width) × 8 m (length) (=27 m^2^), with rows spaced by 0.9 m and plots were separated by 2 m terraces to prevent contamination by superficial run-off containing bacteria, metabolites or fertilizers. The experiments were set in a complete randomized block design with six replicates.

Cultural and phytosanitary managements followed the technical recommendations for the maize crop (Embrapa 2011). The experiments were not irrigated.

### Plant sampling, harvesting and analyses

Between 29 and 57 days after sowing (DAS), depending on the climatic conditions, five plants were randomly collected from each plot to evaluate the performance at the vegetative growth. Shoots were washed and dried to constant weight at 50 °C for evaluation of shoot dry weight (SDW). Shoots were then ground (20 mesh) and total N was determined by sulfuric digestion followed by semi-micro Kjeldahl distillation method, as described before (Hungria et al. 2015a).

At the time of physiological maturity, plant height (PH) was determined based on the average of six plants, and plant population was also estimated. Grain yields (kg ha^−1^) were determined by harvesting a 6.3 m^2^ area (0.9 m wide × 7 m long) from the central portion of each plot. Grains were cleaned and weighed, the humidity evaluated in a grain moisture tester and the content corrected to 13 % moisture. In 2013/2014 and 2014/2015 the N content of seeds was also determined, as described for shoots.

It is worth mentioning that all field experiments were performed according to the Brazilian legislation required for the registration of commercial inoculants or technologies of inoculation for plant growth-promoting bacteria (MAPA 2011).

### Statistical analyses

Data from each experiment were first submitted to tests of normality and homogeneity of variances for each variable and then to the analysis of variance (ANOVA). When significant differences were detected by the F test, Duncan’s multiple-range test at *p* ≤ 0.05 and 0.10 (for inoculant products the Brazilian legislation accepts *p* ≤ 0.10; MAPA 2011) was used as a multiple comparisons procedure.

## Results

In the 2012/2013 crop season, in Ponta Grossa, grain yield of maize plants inoculated with *A. brasilense* and supplied with enriched metabolites either at sowing or at the V3 stage was significantly higher than the other treatments (Table 2). In Cachoeira Dourada, shoot dry weight (SDW) and total N accumulated in shoots (TNS) were significantly increased when *Azospirillum* was inoculated on the seeds (Table 2). The supplementation with enriched metabolites associated to seed inoculation with *Azospirillum* seemed to improve the same parameters at this site, even though this treatment did not show significantly higher SDW than the other treatments (Table 2). In addition, in Cachoeira Dourada both treatments with *Azospirillum* inoculated on seeds along with enriched metabolites, and *Azospirillum*-inoculated by leaf spray added of enriched metabolites promoted higher yield than the other treatments (Table 2).

**Table 2:** Effect of *Azospirillum brasilense* strains CNPSo 2083 and CNPSo 2084 and of enriched metabolites of *R. tropici* strain CIAT 899 applied to the seeds at sowing or by leaf spray at the V3 stage on plant growth (shoot dry weight, SDW; plant height, PH), shoot N (content [NS] and total N accumulated in shoots [TNS]) at 57 and 51 days after sowing (DAS), and grain (yield) at the physiological maturity of maize hybrid DOW 2B 707 HX

Treatment	Ponta Grossa					Cachoeira Dourada				
	57 DAS				Maturity	51 DAS				Maturity
	SDW (g pl^−1^)	PH (cm)	NS (g kg^−1^)	TNS (mgN pl^−1^)	Yield (kg ha^−1^)	SDW (g pl^−1^)	PH (cm)	N (g kg^−1^)	TNS (mgN pl^−1^)	Yield (kg ha^−1^)
Non-inoculated control	56.1^ns^	252^ns^	23.04^ns^	1292^ns^	8406 b	33.1 c	234^ns^	21.16^ns^	700 b	6310 b
Seed inoculated (*Azospirillum*)	58.3	260	23.86	1391	8850 b	44.1 a	242	21.00	926 a	6567 b
Seed inoculated (*Azospirillum* + enriched metabolites)	52.7	253	22.64	1193	9225 a	40.8 ab	243	21.94	895 a	7373 a
Leaf spray inoculation (*Azospirillum*)	55.0	258	22.45	1235	8567 b	36.9 bc	241	21.10	779 b	6543 b
Leaf spray inoculation (*Azospirillum* + enriched metabolites)	55.2	256	22.71	1254	9256 a	36.4 bc	238	21.06	766 b	7286 a

Field experiments performed in Ponta Grossa and Cachoeira Dourada, Brazil, in the summer crop season of 2012/2013. Means (n = 6) on the same column which are followed by different letters are significantly different (*p* ≤ 0.10, Duncan test)

*n.s.* statistically non-significant

In 2013/2014, in Rio Verde, the supplementation of both treatments that received *Azospirillum* inoculation, either on seeds or by leaf spray, with enriched metabolites resulted in significant increases in the N content of the grains (TNG, Table 3). In Maracaí, the best performance was achieved again in the treatment pulverized with *A. brasilense* supplied with enriched metabolites, resulting in greater yield, values of accumulation of N in grains (NG) and TNG, in general statistically higher than all other treatments (Table 3).

**Table 3:** Effect of *Azospirillum brasilense* strains CNPSo 2083 and CNPSo 2084 and of enriched metabolites of *R. tropici* strain CIAT 899 enriched in LCOs applied to the seeds at sowing or by leaf spray at the V3 stage on plant growth (shoot dry weight, SDW; plant height, PH), shoot N (content [NS] and total N accumulated in shoots [TNS]) at 32 and 42 days after sowing (DAS), and grain parameters (yield, N content in grains [NG] and total N accumulated in grains [TNG]) in maize hybrid DKB- 350-PRÓ

Treatment	Rio Verde							Maracaí						
	32 DAS				Maturity			42 DAS				Maturity		
	SDW (g pl^−1^)	PH (cm)	NS (g kg^−1^)	TNS (mgN pl^−1^)	Yield (kg ha^−1^)	NG (g kg^−1^)	TNG (mgN pl^−1^)	SDW (g pl^−1^)	PH (cm)	N (g kg^−1^)	TNS (mgN pl^−1^)	Yield (kg ha^−1^)	NG (g kg^−1^)	TNG (mgN pl^−1^)
Non-inoculated control	13.0^ns^	176^ns^	25.27^ns^	328^ns^	6465^ns^	11.77^ns^	76 b	40.3^ns^	153^ns^	22.49^ns^	806 b	3091 d	14.13 bc	44 c
Seed inoculated (*Azospirillum*)	12.1	174	26.74	324	6758	11.23	76 b	40.4	149	23.41	946 a	3486 bc	15.41 ab	54 b
Seed inoculated (*Azospirillum* + enriched metabolites)	11.9	175	26.81	320	6769	13.93	94 a	40.8	151	23.35	953 a	3698 ab	14.41 abc	53 b
Leaf spray inoculation (*Azospirillum*)	11.8	176	28.08	331	6621	11.12	74 b	40.7	150	23.31	948 a	3278 cd	13.59 c	44 c
Leaf spray inoculation (*Azospirillum* + enriched metabolites)	11.9	174	28.18	335	6792	13.92	94 a	43.7	149	23.39	1022 a	3958 a	15.79 a	62 a

Field experiments performed in Rio Verde and Maracaí in the summer crop season of 2013/2014. Means (n = 6) on the same column which are followed by different letters are significantly different (*p* ≤ 0.10, Duncan test)

*n.s* statistically non-significant

In Londrina, in 2014/2015, leaf spray with *Azospirillum* resulted in significant increases in SDW relative to the non-inoculated controls, either in the presence or in absence of enriched metabolites (Table 4). Seed inoculation resulted in increased grain yield relative to the non-inoculated control, but no further increases were observed when metabolites were added; in contrast, leaf spray inoculation only resulted in yield increases when supplemented with enriched metabolites (Table 4). In Ponta Grossa, the addition of the enriched metabolites to both inoculation with *Azospirillum* by seeds or leaf spray resulted in increased grain yield when compared to the non-inoculated control and to the treatments inoculated only with *Azospirillum* (Table 4).

**Table 4:** Effect of *Azospirillum brasilense* strains CNPSo 2083 and CNPSo 2084 and of metabolites of *R. tropici* strain CIAT 899 enriched with LCOs applied to the seeds at sowing or by leaf spray at the V3 stage on plant growth (shoot dry weight, SDW; plant height, PH), shoot N (content [NS] and total N accumulated in shoots [TNS]) at 32 and 42 days after sowing (DAS), and grain parameters (yield, N content in grains [NG] and total N accumulated in grains [TNG]) in maize hybrid DKB- 350-PRÓ2

Treatment	Londrina							Ponta Grossa						
	29 DAS				Maturity			53 DAS				Maturity		
	SDW (g pl^−1^)	PH (cm)	NS (g kg^−1^)	TNS (mgN pl^−1^)	Yield (kg ha^−1^)	NG (g kg^−1^)	TNG (mgN pl^−1^)	SDW (g pl^−1^)	PH (cm)	NS (g kg^−1^)	TNS (mgN pl^−1^)	Yield (kg ha^−1^)	NG (g kg^−1^)	TNG (mgN pl^−1^)
Non-inoculated control	7.5 b	233^ns^	31.78^ns^	238^ns^	8624 b	15.47^ns^	133 c	42.2^ns^	248^ns^	30.74^ns^	1297^ns^	7723 b	11.86^ns^	92 ^ns^
Seed inoculated (*Azospirillum*)	7.9 ab	236	31.32	247	9472 a	15.76	149 abc	41.0	246	30.70	1259	7632 b	12.17	93
Seed inoculated (*Azospirillum* + enriched metabolites)	7.8 ab	239	32.17	251	9581 a	15.78	151 ab	42.2	249	30.33	1280	8453 a	12.18	103
Leaf spray inoculation (*Azospirillum*)	8.3 a	228	32.32	268	8815 b	15.43	136 bc	40.8	248	30.91	1261	7426 b	12.31	91
Leaf spray inoculation (*Azospirillum* + enriched metabolites)	8.3 a	238	32.43	269	9426 a	16.50	156 a	45.9	256	30.47	1398	8250 a	12.11	100

Field experiments performed in Londrina and Ponta Grossa in the summer crop season of 2014/2015. Means (n = 6) on the same column which are followed by different letters are significantly different (*p* ≤ 0.10, Duncan test)

*n.s* statistically non-significant

## Discussion

One of the main goals of new biotechnological products is to reduce the agricultural utilization of pesticides and/or chemical fertilizers, providing higher sustainability associated with enhanced environmental quality (Hameeda et al. 2006). In this study, we observed that when maize seeds were inoculated with *A. brasilense* strains CNPSo 2083 and CNPSo 2084, there were no increases in grain yield in the leaf spray treatment. When *Azospirillum* was applied on seeds, statistically significant increases were observed in two out of six experiments. Although this percentage is lower than usually reported (Okon and Labandera-Gonzalez 1994; Díaz-Zorita and Fernandez-Canigia 2009; Hungria et al. 2010), increments in yield were observed in all trials, and when a combined analysis was performed, there was a statistically significant gain of 358 kg ha^−1^ in relation to the non-inoculated treatment. However, when the *Azospirillum* inoculant was supplemented with LCO-enriched metabolites from *R. tropici* strain CIAT 899, either by seed inoculation or by leaf spray, statistically significant increases in grain yield in comparison to the non-inoculated control were observed in five out of six field experiments, and when compared to the single inoculation with *Azospirillum*, in three and five out of six experiments for seeds and leaf spray, respectively.

The beneficial relationships between PGPB such as *Azospirillum*, and several plant species have been previously described (Okon, and Labandera-Gonzalez 1994; Bashan and de Bashan 2010; Cassán et al. 2013). Field experiments have shown increases in grain yield ranging from 5 to 75 % (Okon and Labandera-Gonzalez 1994; Fuentes-Ramirez and Caballero-Mellado 2005; Castro-Sowinski et al. 2007; Rodrigues et al. 2008; Hungria et al. 2010). These increases are commonly attributed to root growth promotion, accomplished by phytohormones produced by the bacterium, with an emphasis on indole acetic acid, gibberellins and cytokinins (Tien et al. 1979). Moreover, it is inferred that the application of *Azospirillum* is also responsible for higher rates of absorption of water and minerals by the plant (Okon, and Kapulnik 1986; Dardanelli et al. 2008) and higher tolerance to abiotic stresses, such as drought and salinity (Cassán et al. 2009; Zawonski et al. 2011).

The relationship between different soil microorganisms and the role of metabolites secreted by them on growth of other surrounding microbial species and plants has been the subject of numerous studies. For example, Massoud et al. (2009) studied the effects of the combined inoculation of mycorrhizal fungi, *Bacillus circulans, Rhizobium* sp., *Azospirillum lipoferum, Azotobacter chroococcum* and mineral rocks on common bean (*Phaseolus vulgaris* L.) plants. The inoculum consortium promoted higher nitrogenase activity and increased the availability of macronutrients, besides promoting plant growth, resulting in increased yield in comparison to the single inoculation (Massoud et al. 2009). The authors attributed these results at least partially to the exudation of beneficial molecules by the microorganisms (Massoud et al. 2009). The positive effects of molecules such as LCOs, exopolysaccharides (EPSs), and plant hormones on plant growth (hosts or non-hosts) may be associated with increased survival and capacity of plant infection by beneficial rhizospheric bacteria and fungi and/or with plant growth promotion (Marks et al. 2013). In a study with the legume model *Medicago truncatula*, application of LCOs of *Sinorhizobium meliloti* facilitated root infection by mycorrhizal fungi and stimulated lateral root hair development (Olah et al. 2005). It is possible that LCOs, although produced by rhizobia, have a direct influence on the rhizospheric microbial community by influencing interactions among microorganisms and promoting plant growth, including growth of non-host plants.

The LCOs secreted by rhizobia are described as responsible for several physiological modifications in the root hairs of legumes. Such changes include alterations in ion flux, membrane depolarization of root cells, intra and extracellular alkalization, synthesis of phosphatidic acid and diacylglycerol, accumulation of reactive oxygen species, root hair deformations involving changes in actin cytoskeleton, cell division activation and induction of the expression of genes involved in nodulation (Mulder et al. 2006; Cooper 2007). All these changes allow the rhizobia to invade the host plant, leading to the formation of nodule primordia (Riely et al. 2004), and therefore, LCOs would behave as mitogenic and morphogenic agents (Rélic et al. 1993). However, intriguingly, LCOs have also been described as growth regulators of a wide variety of non-leguminous plants (Prithiviraj et al. 2003), inducing various physiological responses (Souleimanov et al. 2002), as increased seed germination, lateral root development and nutrient uptake (Smith et al. 2015). The study by Rélic et al. (1993) supports the hypothesis that the LCOs may act as plant hormones when applied to non-host plants. Previous studies with cells and plants of tobacco (*Nicotiana* sp.) (Baier et al. 1999), tomato (*Solanum lycopersicum* L.) (Staehelin et al. 1994) and carrot (*Dacus carota* L.) (De Jong et al. 1993) have shown that LCOs are activators of cell division and embryonic development of non-host plants.

Marks et al. (2013) observed, in previous field experiments, an 11.4 % increase in the grain yield of maize inoculated with the same strains of *A. brasilense* and supplied with concentrated metabolites of *R. tropici* that included LCOs. Although the mechanisms responsible for the benefits of LCOs in non-leguminous are not fully understood, the application of such molecules must somehow modify the hormone balance, affecting plant growth and development (Souleimanov et al. 2002). The most effective contribution of LCOs to non-leguminous plants might be the stimulation of root development, increasing the absorption of water and nutrients and resulting in improved plant growth and yields (Smith et al. 2015). In the field experiments performed in our study, the application of LCOs-rich rhizobial metabolites seems to have affected the N metabolism, increasing the N content of shoots and grains, and also influencing grain yield.

Our results have also shown that the application of LCOs-rich rhizobial metabolites by leaf spray resulted in higher grain yields. Khan (2003) also observed that the leaf application of LCOs in maize stimulated photosynthesis, increased leaf area and shoot dry weight. In another study, Chen et al. (2006) applied LCOs of *Bradyrhizobium japonicum* to tomato leaves and observed the anticipation of flowering and fruiting and an increase in the number and weight of fruits under greenhouse conditions, as well as a 30 % increase in the number and fruit weight in a field experiment. The benefits of LCOs leaf spray can be attributed to the fact that these molecules indirectly affect the photosynthesis and accelerate growth, probably by the stimulation of mitotic activity in meristematic tissue of leaves (Khan et al. 2008). It can also be inferred that the foliar application of LCOs promotes the suppression of innate immune responses, which possibly facilitates the microbial interactions, such as the invasion and colonization by endophytic bacteria (Liang et al. 2013).

The rationale of the utilization of metabolites of *R. tropici* CIAT 899 enriched on LCOs was based on some interesting properties of this strain, which produces a broad variety of LCOs, even in the absence of inducing flavonoids, when subjected to abiotic stresses such as acidity (Morón et al. 2005) and salinity (Estévez et al. 2009; del Cerro et al. 2015a, b). *R. tropici* carries five copies of *nod* gene (Ormeño-Orrillo et al. 2012) and, recently, the synthesis of several LCOs structures related to *nodD1,nodD2, nodD3, nodD4* and *nodD5* genes has been elucidated (del Cerro et al. 2015a, b). The production of a large variety of LCOs by *R. tropici* CIAT 899 may represent a strategy for nodulation of several host plants under various environmental stressful conditions (Liang et al. 2013; del Cerro et al. 2015a, b). Consequently, it is possible that these LCOs also favor the systemic resistance, particularly in leaf spray, giving greater vigor to the plants, and resulting in increases in crops yields. Therefore, it is likely that LCOs have a broad spectrum of action in regulating plant growth, in addition to its primary function in nodulation of legumes.

The results of our study reveal the biotechnological potential of adding microbial metabolites, in our case rhizobial metabolites enriched with LCOs to products for leaf spray and seed inoculation of non-leguminous plants, such as maize. This knowledge can be applied to the improvement of commercial products, taking into account the need for developing a new generation of inoculants carrying microorganisms and microbial metabolites.
